# Tumor B7-H3 (CD276) Expression and Survival in Pancreatic Cancer

**DOI:** 10.3390/jcm7070172

**Published:** 2018-07-10

**Authors:** Kentaro Inamura, Yutaka Takazawa, Yosuke Inoue, Yusuke Yokouchi, Maki Kobayashi, Akio Saiura, Tomoko Shibutani, Yuichi Ishikawa

**Affiliations:** 1 Division of Pathology, The Cancer Institute, Department of Pathology, The Cancer Institute Hospital, Japanese Foundation for Cancer Research, 3-8-31 Ariake, Koto-ku, Tokyo 135-8550, Japan; kentaro.inamura@jfcr.or.jp (K.I.); maki.kobayashi@jfcr.or.jp (M.K.); ishikawa@jfcr.or.jp (Y.Is.); 2 Department of Hepato-Biliary-Pancreatic Surgery, The Cancer Institute Hospital, Japanese Foundation for Cancer Research, Tokyo 135-8550, Japan; yosuke.inoue@jfcr.or.jp (Y.I.); akio.saiura@jfcr.or.jp (A.S.); 3 Biomarker Department, Daiichi Sankyo Co., Ltd., Tokyo 140-8710, Japan; lalalagamy@nifty.com (Y.Y.); shibutani.tomoko.zc@daiichisankyo.co.jp (T.S.)

**Keywords:** B7-H3, clinical outcome, immune checkpoint inhibitor, immune modulator, immunotherapy, pancreatic ductal adenocarcinoma, prognosis, tumor microenvironment

## Abstract

B7-H3 (CD276), a member of the family of immune modulators, orchestrates antitumor immunity. To date, only small-sized studies have examined the association of B7-H3 expression with survival in pancreatic cancer, yielding inconclusive results. We evaluated tumor B7-H3 expression in 150 consecutive patients with pancreatic ductal adenocarcinoma using immunohistochemistry. B7-H3 expression was positive (≥10% tumor cells) in 99 of 150 (66%) cases of pancreatic cancer. We classified the tumors into four groups depending on B7-H3 expression (negative, low, intermediate, and high) and found that higher B7-H3 expression was independently associated with lower disease-free survival (DFS; for high vs. negative B7-H3 expression: multivariable hazard ratio (HR) = 3.12; 95% confidence interval (CI) = 1.48–6.15; *P*_trend_ = 0.0026). Furthermore, the association of B7-H3 expression with survival differed according to the pathological stage (p-stage) (*P*_interaction_ = 0.048, between p-stages I–II and III–IV). The association of B7-H3 positivity with lower DFS was stronger in tumors with p-stage I–II (multivariable HR = 3.10, 95% CI = 1.75–5.69; *P* < 0.0001) than in those with p-stage III–IV (multivariable HR = 1.20, 95% CI = 0.67–2.28; *P* = 0.55). We demonstrated that tumor high B7-H3 expression is independently associated with poor survival in patients with pancreatic cancer and that this association is stronger in tumors with p-stage I–II than in those with p-stage III–IV. B7-H3 expression may be a useful prognostic biomarker for identifying aggressive early-stage pancreatic cancer.

## 1. Introduction

Pancreatic cancer is one of the most life-threatening malignancies, with the highest mortality among all solid tumors [1,2]. It is characterized by an abundant stromal component that comprises up to 90% of its total volume [2,3,4,5,6,7,8,9,10]. The tumor stroma contains a highly immunosuppressive microenvironment resulting from the crosstalk between tumor and immune cells, which promotes oncogenesis and tumor progression [2,3,4,5,6,7,8,9,10]. Emerging immunotherapies, including anti-PD-1 (PDCD1)/PD-L1 (CD274) antibodies, have been used effectively to treat several types of cancers, including non-small-cell lung cancer (NSCLC) and malignant melanoma [11,12]; however, these therapies lack efficacy in treating pancreatic cancers, partly due to their abundant stroma with an immunosuppressive microenvironment [2,3,4,5,6,7,8,9,10,13,14,15,16,17,18]. To improve the outcomes in patients with pancreatic cancer, it is critical to identify the mechanisms underlying the tumor microenvironment as well as new molecular targets for the development of effective strategies against this fatal cancer type [2,3,4,5,6,7,8,9,10].

B7-H3 (CD276) belongs to a family of immune modulators that also includes PD-L1 (also known as B7-H1) [19,20,21]. Recent evidence demonstrates that B7-H3 blockade, especially when combined with an anti-PD-1 blockade, is a promising strategy for the treatment of B7-H3-expressing NSCLCs that are refractory to anti-PD-1 therapy [22], making B7-H3 a promising molecular target [22,23,24,25,26,27,28]. However, the association of B7-H3 expression with survival in patients with pancreatic cancer remains elusive. Several reports have examined this association, but the sample sizes were small and the results were inconclusive [29,30,31,32,33,34,35]. In addition, no study has examined whether the association of B7-H3 expression with survival differs according to clinicopathological variables in pancreatic cancer.

Therefore, we aimed to examine the association of B7-H3 expression with survival in pancreatic cancer using 150 consecutive pancreatic cancer cases. Furthermore, we examined whether the association of B7-H3 expression with patient mortality differed according to clinicopathological variables.

## 2. Materials and Methods

### 2.1. Patients

We enrolled 150 consecutive cases of pancreatic ductal adenocarcinoma based on the availability of the tumor B7-H3 expression status, survival and recurrence. The patients were Japanese and had undergone tumor resection between March 2005 and December 2011 at The Cancer Institute Hospital, Japanese Foundation for Cancer Research (JFCR), Tokyo, Japan. We excluded patients who underwent preoperative therapy. We monitored the patients until death or December 2016. All patients were pathologically staged according to the 8^th^ edition of the AJCC staging system [36]. T4 was defined as tumors with the radiologically confirmed abutment of celiac artery, common hepatic artery, or superior mesenteric artery at the preoperative multidisciplinary team conference because T4 cannot be determined only by pathological findings. All patients included in this study provided informed consent for research, and the study plan was approved by the institutional review board of JFCR.

### 2.2. B7-H3 Immunohistochemistry

B7-H3 expression was immunohistochemically evaluated in cancer cells using tissue microarrays (TMAs). Using archived surgically resected specimens which had been used for the initial pathological diagnosis of primary pancreatic cancer, we constructed TMAs as previously described [37]. Briefly, we punched selected regions of the donor paraffin blocks with a coring needle of 2-mm diameter and transferred the material to the array in the recipient block using a manual tissue arrayer (KIN-1; Azumaya, Tokyo, Japan). For each tumor, an experienced pathologist (Y.T.) selected areas from the tumor center and periphery based on spatial heterogeneity.

B7-H3 immunostaining was performed as previously described [38]. Briefly, 4-μm thick sections were immunohistochemically stained for B7-H3 with an anti-B7-H3 mouse monoclonal antibody (1:400; clone: BD/5A11; Daiichi Sankyo Co., Ltd., Tokyo, Japan) using the Leica Bond III automated system (Leica Biosystems Melbourne Pvt., Ltd., Melbourne, Australia). The sections were incubated at pH 9 for 10 min at 100 °C. For positive and negative controls, we used a B7 subfamily cell array (Daiichi Sankyo Co., Ltd.), consisting of CHO-K1 cells overexpressing the B7 subfamily members as previously described [39]. The CHO-K1 cells, which transiently overexpressed B7-H1, B7-H2, B7-H3, B7-H4, B7-1 or B7-2, and mock-transfected control cells, were fixed in 10% neutral-buffered formalin and paraffin embedded to obtain cell block arrays. Using the B7 subfamily cell array, we also verified the specificity and sensitivity of the anti-B7-H3 antibody (clone: BD/5A11).

**Figure 1:**
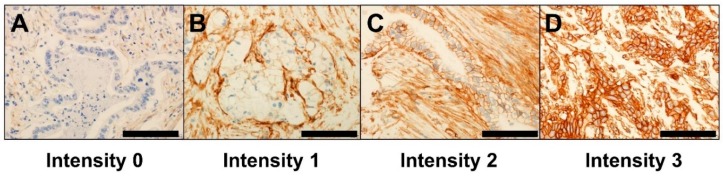
Immunohistochemical staining of B7-H3 in tumor membranes of pancreatic cancer. (**A**) Intensity 0 (absent), (**B**) intensity 1 (weak), (**C**) intensity 2 (intermediate), and (**D**) intensity 3 (strong). Scale bar, 100 µm.

Expression levels of B7-H3 (B7-H3 intensity) in cancer cell membranes were defined as 0 (absent), 1 (weak), 2 (intermediate), or 3 (strong), as shown in Figure 1. Because B7-H3 expression was also observed in stromal cells, we evaluated B7-H3 expression only in tumor cells. The percentage of tumor cells at each B7-H3 intensity was evaluated. Based on the staining intensity and percentage of positive tumor cells, we divided the specimens into two groups: B7-H3-negative (intensity 1 or more <10%) and -positive (intensity 1 or more ≥10%) groups. Furthermore, we classified the B7-H3-positive group into three subgroups: B7-H3 low (intensity 1 or more ≥10% and intensity 2 or more <10%), intermediate (intensity 2 or more ≥10% and intensity 3 <10%), and high (intensity 3 ≥10%) groups. The expression of B7-H3 was interpreted by an experienced pathologist (K.I.) who was blinded to the data. All the samples were blindly examined by a second pathologist (Y.Y.). The agreement between the two pathologists for B7-H3 expression in tumor membranes was good, with a kappa of 0.78 (*P* < 0.0001) for the two groups and weighted kappa of 0.78 (*P* < 0.0001) for the four subgroups, indicating substantial agreement.

### 2.3. Statistical Analysis

All statistical analyses were conducted using JMP 12 software (SAS Institute Inc., Cary, NC, USA). All *P* values were two-sided. We considered *P* < 0.05 as statistically significant. *P* values were interpreted very cautiously by considering multiple hypothesis testing. The kappa coefficient was calculated to assess the level of agreement between the two pathologists regarding the results of the immunohistochemical analyses. To investigate the associations of tumor B7-H3 positivity with different clinicopathological factors, we performed the chi-square test. The survival duration was defined as the interval between the date of surgery and the last follow-up or death. For survival analysis, the Kaplan-Meier method was used to assess the survival time distribution according to B7-H3 expression status, whereas the log-rank test was used to test any significant deviation from the null hypothesis. For analyses of disease-free survival (DFS), deaths resulting from other causes were censored. We used univariable and multivariable Cox proportional hazards regression models to calculate the hazard ratios (HRs) and 95% confidence intervals (CIs) for mortality according to the B7-H3 expression status. The multivariable model initially included age (≤65 vs. >65 years), sex (male vs. female), body-mass index (<25 vs. ≥25 kg/m^2^), preoperative CEA levels (≤5 vs. >5 ng/mL), preoperative CA19-9 levels (<500 vs. ≥500 U/mL), tumor location (head vs. body/tail), pathological stage (p-stage) (I–II vs. III–IV)., and adjuvant chemotherapy (present vs. absent). A backward stepwise elimination with *P* equal to 0.20 as the threshold was performed to select variables for the final model. *P* values for interaction between tumor B7-H3 positivity and different clinicopathological factors were assessed using the Wald test on the cross-product of B7-H3 expression (negative vs. positive) and stratification valuable in the Cox model. We confirmed the proportionality of hazards assumption using the graphs of the log[−log(survival probability)] vs. log of survival time to visually assess whether the lines were approximately parallel.

## 3. Results

### 3.1. Tumor B7-H3 Expression in Pancreatic Cancer

Of the 150 pancreatic cancer cases, 99 (66%) were B7-H3-positive (Figure 1). The classification of tumors based on B7-H3 expression level revealed that the B7-H3 negative, low, intermediate, and high groups comprised 51 (34%), 67 (45%), 20 (13%), and 12 (8%) cases, respectively. The clinicopathological characteristics of the pancreatic cancer cases are summarized in Table 1. B7-H3 positivity was not associated with any variables examined (*P* ≥ 0.11).

### 3.2. B7-H3 Expression Status and Survival in Patients with Pancreatic Cancer

Among the 150 patients, there were 127 deaths, including 122 disease-specific deaths, during a median follow-up period of 20 months (interquartile range: 11–38 months). The 2-/5-year DFS and overall survival were 23%/14% and 42%/15%, respectively.

**Figure 2:**
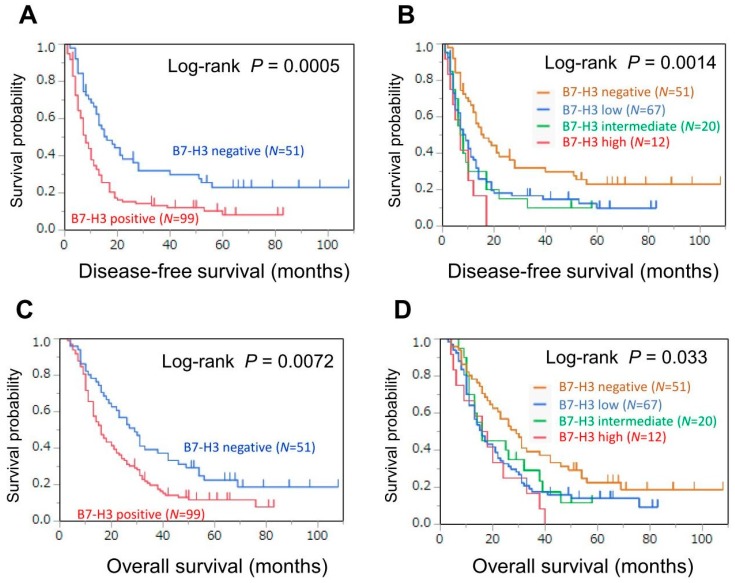
Kaplan-Meier curves for disease-free (**A**,**B**) and overall survival (**C**,**D**) in patients with pancreatic cancer (**A**,**C**) according to tumor B7-H3 expression (negative or positive) or (**B**,**D**) according to tumor B7-H3 expression (negative, low, intermediate, or high).

We assessed the association of B7-H3 negativity or positivity with DFS and overall survival. In the Kaplan-Meier analysis, the 2-/5-year DFS was 38%/23% in patients with B7-H3-negative tumors and 15%/8% in patients with B7-H3-positive tumors (log-rank *P* = 0.0005; Figure 2A). The 2-/5-year overall survival was 57%/23% in patients with B7-H3-negative tumors and 34%/12% in patients with B7-H3-positive tumors (log-rank *P* = 0.0072; Figure 2C). In the multivariable Cox regression analysis, compared with B7-H3-negative tumors, B7-H3-positive tumors were associated with lower DFS (multivariable HR = 1.99, 95% CI = 1.32−3.06; *P* = 0.0009) and lower overall survival (multivariable HR = 1.69, 95% CI = 1.13−2.57; *P* = 0.011; Table 2).

Next, we examined the association of B7-H3 expression levels (negative, low, intermediate, or high) with DFS and overall survival. In the Kaplan-Meier analysis, the 2-year DFS was 38%, 18%, 15%, and 0% in patients with tumors expressing negative, low, intermediate, and high B7-H3 levels, respectively (log-rank *P* = 0.0014; Figure 2B). The 2-year overall survival was 57%, 33%, 45%, and 25% in patients with tumors expressing negative, low, intermediate, and high B7-H3 levels, respectively (log-rank *P* = 0.033; Figure 2D). In more detail, even for patients with low expression of B7-H3 in tumor cells, the survival curve was obviously under the patients with negative expression. In the multivariable Cox regression analysis, higher B7-H3 expression was independently associated with lower DFS (comparing high vs. negative: multivariable HR = 3.12; 95% CI = 1.48–6.15; *P* for trend = 0.0026; Table 3), whereas tumors with higher B7-H3 expression appeared to be associated with lower overall survival (comparing high vs. negative: multivariable HR = 2.21; 95% CI = 1.06−4.28; *P* for trend = 0.055).

### 3.3. Effects of Clinicopathological Variables on B7-H3 Expression and Survival

**Figure 3:**
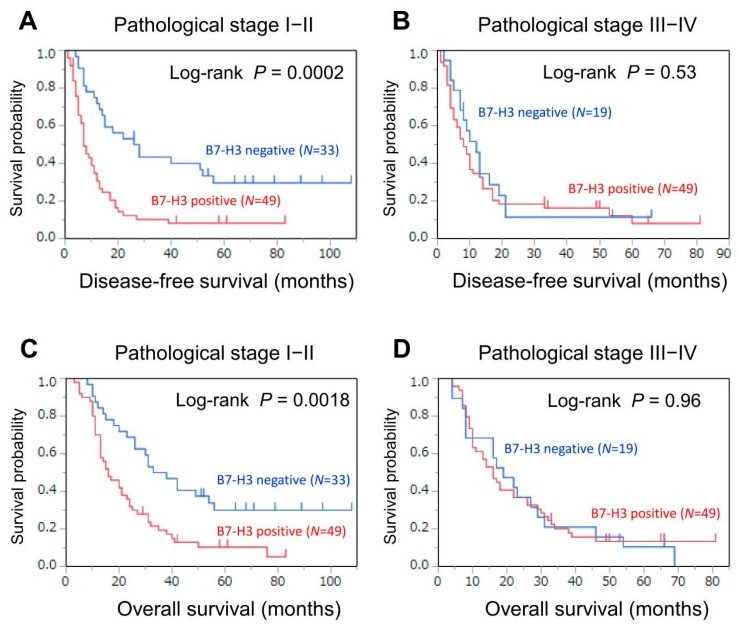
Kaplan-Meier curves for disease-free (**A**,**B**) and overall (**C**,**D**) survival in patients with pancreatic cancer according to tumor B7-H3 expression (negative or positive) in strata of pathological stage. (**A**,**C**) Pathological stage I−II. (**B**,**D**) Pathological stage III−IV.

As our secondary analysis, we examined whether the association of B7-H3 expression with survival was modified by clinicopathological variables (Table 4). We found that the tumor p-stage affected the association between B7-H3 expression and clinical outcome (Table 4). In tumors with p-stage I or II, B7-H3 tumor positivity was associated with lower DFS (log-rank *P* = 0.0002; Figure 3A) and lower overall survival (log-rank *P* = 0.0018; Figure 3C), whereas in tumors with p-stage III or IV, B7-H3 expression was not associated with DFS (log-rank *P* = 0.53; Figure 3B) or overall survival (log-rank *P* = 0.96; Figure 3D). In the multivariable analysis, tumor p-stage affected the association between B7-H3 expression and survival (*P* for interaction = 0.048 for DFS and *P* for interaction = 0.033 for overall survival; Table 4). Among tumors with p-stage I or II, B7-H3 positivity was associated with lower DFS (multivariable HR = 3.10, 95% CI = 1.75–5.69; *P* < 0.0001) and lower overall survival (multivariable HR = 2.59, 95% CI = 1.49−4.67; *P* = 0.0007), whereas in tumors with p-stage III or IV, B7-H3 positivity was not associated with DFS (multivariable HR = 1.20, 95% CI = 0.67−2.28; *P* = 0.55) and overall survival (multivariable HR = 1.03, 95% CI = 0.58−1.88; *P* = 0.93).

## 4. Discussion

We conducted this study to examine the association of tumor B7-H3 expression with clinical outcomes in patients with pancreatic cancer. B7-H3 is a member of the family of immune modulators [19,20,21]; however, its role in the modulation of the tumor microenvironment has not been fully elucidated. Although several studies have examined the association of B7-H3 expression with survival in pancreatic cancer, the results were inconclusive [29,30,31,32,33,34,35]. In this study, using 150 consecutive cases of pancreatic ductal adenocarcinoma, we demonstrated that tumor B7-H3 expression is associated with lower survival and that this association is pronounced in tumors of p-stages I and II. Interestingly, this study suggests a potential role of B7-H3 expression in tumor progression, particularly during the early stages of pancreatic cancer.

Pancreatic ductal adenocarcinoma is a disruptive malignancy and is highly refractory to general therapies [1,2]; furthermore, it is often lethal. Its aggressiveness partially depends on the extent of cancer-associated inflammation in its abundant stroma, which accounts for >90% of the tumor mass [2,3,4,5,6,7,8,9,10]. A marked infiltration of immunosuppressive inflammatory cells into the tumor stroma is considered an early and consistent event in oncogenesis and tumor progression [2,3,4,5,6,7,8,9,10]. From the early stages of tumor initiation, immunosuppressive regulatory T lymphocytes and Gr1^+^CD11b^+^ myeloid-derived suppressor cells are recruited to the tumor stroma, restraining the T-cell-mediated antitumor immunity [40]. The pancreatic tumor microenvironment restricts immune surveillance and promotes tumorigenesis via a crosstalk between tumor and immune cells [2,4]. Emerging evidence indicates that existing immune checkpoint inhibitors, such as anti-PD-1, anti-PD-L1, and anti-CTLA4 antibodies, can be effective monotherapies for immune-sensitive cancers, including NSCLC and malignant melanoma [11,12], but they lack efficacy in the case of pancreatic cancers [2,3,4,5,6,7,8,9,10,13,14,15,16,17,18]. The highly immunosuppressive pancreatic tumor microenvironment is the major reason owing to which this cancer is resistant to immunotherapies [14,15,16,17]. In an era of rapidly evolving immunotherapies, it is critical to identify new molecular targets for the development of effective immunotherapies against the devastating pancreatic cancer [2,3,4,5,6,7,8,9,10].

B7-H3 belongs to the B7 superfamily of type I transmembrane proteins that includes PD-L1 (B7-H1) [19,20,21]. Accumulating evidence demonstrates that B7-H3 orchestrates antitumor immunity by providing co-stimulatory and co-inhibitory signals in the context of different cancers [19,20,21,41]. Recently, immunotherapies have emerged as promising therapeutic strategies against various malignancies; however, most pancreatic cancers are refractory to immune checkpoint inhibitors, possibly because of their highly immunosuppressive microenvironment [14,15,16,17]. Recent evidence demonstrates that B7-H3-expressing NSCLC escapes antitumor immunity via CD8^+^ T-cell repression despite anti-PD-1 immunotherapy and that B7-H3 blockade, especially in combination with the PD-1 blockade, exhibits potent antitumor efficacy mediated by increased number of tumor-specific CD8^+^ T cells [22]. This study represented a novel and promising therapeutic strategy against B7-H3-expressing tumors [22,23,24,25,26,27,28].

Several studies have reported the association of tumor B7-H3 expression with survival in pancreatic cancer; however, the sample sizes were small and the results were inconclusive [29,30,31,32,33,34,35]. Varying results may be a result of limited sample sizes. To the best of our knowledge, our study employed the largest sample size of pancreatic cancer to examine the association of B7-H3 expression with patient mortality. There has been no standardized method to evaluate B7-H3 expression immunohistochemically, which may result in conflicting results of studies on B7-H3 expression. As a validation of the anti-B7-H3 antibody (clone: BD/5A11) used in this study, we confirmed its specificity and sensitivity by using a B7 subfamily cell array that comprises the B7 subfamily (PD-L1 (B7-H1), PD-L2 (B7-DC), B7-H2, B7-H3, B7-H4, B7-1, and B7-2)-overexpressing CHO-K1 cells and mock-transfected control cells. Using the largest sample size and validating B7-H3 immunohistochemistry, our study demonstrated that higher expression of tumor B7-H3 is independently associated with inferior mortality in pancreatic cancer.

No study has examined whether the association of B7-H3 expression with survival differs according to clinicopathological variables. Although both preoperative levels of CA19-9 and adjuvant chemotherapy were independently associated with patient survival, the association of B7-H3 expression with survival did not differ according to their status (data not shown). Interestingly, we have shown that the prognostic association differed according to p-stage. Indeed, the prognostic association was significantly stronger in tumors with p-stage I−II than those with p-stage III−IV. This finding suggests that B7-H3 expression may be a useful prognostic biomarker for identifying aggressive early-stage pancreatic cancer.

We recognize limitations of the current study. First, the observational nature of this study does not allow the determination of causal relationships between high tumor B7-H3 expression and low survival, especially in tumors with p-stages I and II. Nonetheless, experimental evidence suggests a causal association of tumor B7-H3 expression with tumor progression and aggressiveness in pancreatic cancers [29,31,42]. Second, we used TMAs to assess tumor B7-H3 expression. Because of intratumor heterogeneity, a subset of tumors with heterogeneous B7-H3 positivity may have been scored as negative in TMAs. However, this potential misclassification of tumors due to heterogeneity would be expected to be distributed nearly at random, and hence, would most likely have yielded null results. Despite this limitation, we were able to demonstrate the significant association of B7-H3 expression with survival. Third, we evaluated B7-H3 expression only in tumor cells. We also observed B7-H3 expression in stromal cells and substantial differences of stromal B7-H3 expression among cases; however, we evaluated tumor B7-H3 expression without considering stromal expression. It is possible that B7-H3 expression in stromal cells or a combination of tumor and stromal cells might also have a biological or prognostic significance [43,44]. Finally, the findings of this study may not be generalizable because we only enrolled Japanese patients from a single hospital. However, to the best of our knowledge, the sample size of the current study is the largest among the studies that have examined the association of B7-H3 expression with survival in pancreatic cancer. Nonetheless, given a growing interest in racial disparity, additional studies with other races and with larger sample size are warranted to confirm our findings.

In conclusion, we demonstrated that high tumor B7-H3 expression is associated with high mortality in patients with pancreatic cancer and that this association is pronounced during the early stages. Additional studies are needed to confirm our findings and to examine the signaling pathways involved and potential for interventions targeting B7-H3 in patients with early-stage pancreatic cancer.

## Figures and Tables

**Table 1:** Clinicopathological and molecular characteristics of pancreatic cancer. According to tumor B7-H3 expression (negative vs. positive).

Variables		*N* of Samples (%)	B7-H3 Expression		
			Negative *N* = 51 (34%)	Positive *N* = 99 (66%)	*P* Values
Age					1.00
	≤65 years	60 (40%)	20 (39%)	40 (40%)	
	>65 years	90 (60%)	31 (61%)	59 (60%)	
Gender					0.22
	Male	89 (59%)	34 (67%)	55 (56%)	
	Female	61 (41%)	17 (33%)	44 (44%)	
Body-mass index					0.78
	<25 kg/m^2^	134 (89%)	45 (88%)	89 (90%)	
	≥25 kg/m^2^	16 (11%)	6 (12%)	10 (10%)	
CEA					0.85
	≤5 ng/mL	112 (75%)	39 (76%)	73 (74%)	
	>5 ng/mL	38 (25%)	12 (24%)	26 (26%)	
CA19-9					0.50
	<500 U/mL	88 (68%)	29 (64%)	59 (70%)	
	≥500 U/mL	41 (32%)	16 (36%)	25 (30%)	
Tumor location					0.49
	Head	87 (58%)	32 (63%)	55 (56%)	
	Body/tail	63 (42%)	19 (37%)	44 (44%)	
Pathological stage					0.11
	I–II	82 (55%)	33 (63%)	49 (50%)	
	III–IV	68 (45%)	19 (37%)	49 (50%)	
Adjuvant chemotherapy					0.73
	Absent	32 (22%)	10 (20%)	22 (22%)	
	Present	116 (78%)	40 (80%)	76 (78%)	

**Table 2:** B7-H3 expression (negative and positive) and patient survival * in pancreatic cancer.

	Disease-Free Survival				Overall Survival			
	Univariable Analysis		Multivariable Analysis **		Univariable Analysis		Multivariable Analysis **	
	HR (95% CI)	*P* Values	HR (95% CI)	*P* Values	HR (95% CI)	*P* Values	HR (95% CI)	*P* Values
B7-H3: positive (vs. negative)	1.91 (1.31–2.84)	0.0006	1.99 (1.32–3.06)	0.0009	1.66(1.14–2.44)	0.0074	1.69 (1.13–2.57)	0.011
Adjuvant chemotherapy: absent (vs. present)	1.94 (1.26–2.90)	0.0032	2.10 (1.32–3.25)	0.0021	1.75 (1.14–2.62)	0.012	1.77 (1.11–2.75)	0.018
CA19-9 (U/mL): ≥500 (vs. <500)	1.85 (1.23–2.75)	0.0034	1.86 (1.23–2.77)	0.0036	1.67 (1.11–2.47)	0.014	1.67 (1.11–2.48)	0.016
Pathological stage: III−IV (vs. I−II)	1.30 (0.91–1.85)	0.14			1.38 (0.97–1.95)	0.075	1.29 (0.87–1.90)	0.20
CEA (ng/mL): >5 (vs. ≤5)	1.41 (0.94–2.08)	0.093			1.41 (0.92–2.08)	0.10		
Gender: female (vs. male)	1.26 (0.88–1.80)	0.20			1.21 (0.85–1.71)	0.30		
Body-mass index (kg/m^2^): ≥25 (vs. <25)	1.27 (0.71–2.12)	0.40			1.13 (0.63–1.88)	0.66		
Age (years): ≤65 (vs. >65)	1.09 (0.76–1.56)	0.63			1.02 (0.71–1.45)	0.93		
Location of primary tumor: body/tail (vs. head)	1.02 (0.71–1.45)	0.91			1.00 (0.70–1.42)	1.00		

* Cox proportional hazards regression models were used to calculate the HR and 95% CI. ** The multivariable model initially included age (≤65 vs. >65 years), sex (male vs. female), body-mass index (<25 vs. ≥25 kg/m^2^), preoperative CEA levels (≤5 vs. >5 ng/mL), preoperative CA19-9 levels (<500 vs. ≥500 U/mL), tumor location (head vs. body/tail), pathological stage (I−II vs. III−IV), and adjuvant chemotherapy (present vs. absent). A backward stepwise elimination with *P* equal to 0.20 as the threshold was performed to select variables for the final model. CI, confidence interval; HR, hazard ratio.

**Table 3:** B7-H3 expression (negative, low, intermediate, and high) and patient survival * in pancreatic cancer.

	Disease-Free Survival				Overall Survival			
	Univariable Analysis		Multivariable Analysis **		Univariable Analysis		Multivariable Analysis **	
	HR (95% CI)	*P* Values	HR (95% CI)	*P* Values	HR (95% CI)	*P* Values	HR (95% CI)	*P* Values
B7-H3 negative (*N* = 51)	1 (referent)		1 (referent)		1 (referent)		1 (referent)	
B7-H3 low (*N* = 67)	1.78 (1.18–2.70)	0.0056	1.79 (1.14–2.82)	0.011	1.63 (1.09–2.46)	0.017	1.64 (1.05–2.59)	0.028
B7-H3 intermediate (*N* = 20)	1.97 (1.10–3.42)	0.024	2.41 (1.26–4.40)	0.0092	1.49 (0.82–2.59)	0.19	1.66 (0.86–3.07)	0.12
B7-H3 high (*N* = 12)	2.97 (1.47–5.59)	0.0035	3.12 (1.48–6.15)	0.0039	2.21 (1.10–4.11)	0.027	2.21 (1.06–4.28)	0.036
*P* values for trend		0.044		0.0026		0.040		0.055

* Cox proportional hazards regression models were used to calculate the HR and 95% CI. ** The multivariable model initially included age (≤65 vs. >65 years), sex (male vs. female), body-mass index (<25 vs. ≥25 kg/m^2^), preoperative CEA levels (≤5 vs. >5 ng/mL), preoperative CA19-9 levels (<500 vs. ≥500 U/mL), tumor location (head vs. body/tail), pathological stage (I−II vs. III−IV), and adjuvant chemotherapy (present vs. absent). A backward stepwise elimination with *P* equal to 0.20 as the threshold was performed to select variables for the final model. CI, confidence interval; HR, hazard ratio.

**Table 4:** B7-H3 expression (negative and positive) in pancreatic cancer and patient survival * in strata of covariates.

		Disease-Free Survival				Overall Survival			
		Univariable Analysis		Multivariable Analysis **		Univariable Analysis		Multivariable Analysis **	
		HR (95% CI)	*P* Values	HR (95% CI)	*P* Values	HR (95% CI)	*P* Values	HR (95% CI)	*P* Values
Pathological stage: I−II	B7–H3 negative	1 (referent)		1 (referent)		1 (referent)		1 (referent)	
(*N* = 93)	B7–H3 positive	2.55 (1.54–4.34)	0.0003	3.10 (1.75–5.69)	<0.0001	2.19 (1.32–3.72)	0.0021	2.59 (1.49–4.67)	0.0007
Pathological stage: III−IV	B7–H3 negative	1 (referent)		1 (referent)		1 (referent)		1 (referent)	
(*N* = 57)	B7–H3 positive	1.19 (0.68–2.18)	0.54	1.20 (0.67–2.28)	0.55	0.99 (0.58–1.76)	0.96	1.03 (0.58–1.88)	0.93
*P* values for interaction***			0.069		0.048		0.045		0.033

* Cox proportional hazards regression models were used to calculate the HR and 95% CI. ** The multivariable model initially included age (≤65 vs. >65 years), sex (male vs. female), body-mass index (<25 vs. ≥25 kg/m^2^), preoperative CEA levels (≤5 vs. >5 ng/mL), preoperative CA19-9 levels (<500 vs. ≥500 U/mL), tumor location (head vs. body/tail), pathological stage (I−II vs. III−IV), and adjuvant chemotherapy (present vs. absent). A backward stepwise elimination with *P* equal to 0.20 as the threshold was performed to select variables for the final model. *** *P* values for interaction between tumor B7-H3 positivity and different clinicopathological factors were assessed using the Wald test on the cross-product of B7-H3 expression (negative vs. positive) and stratification valuable in the Cox model. CI, confidence interval; HR, hazard ratio.

## Supplementary Materials

The following is available online at http://www.mdpi.com/2077-0383/7/7/172/s1, Figure S1: Kaplan-Meier curves for overall survival in patients with pancreatic cancer (**A**) according to tumor B7-H3 expression (negative or positive) or (**B**) according to tumor B7-H3 expression (negative, low, intermediate, or high). Kaplan-Meier curves for overall survival in patients with pancreatic cancer according to tumor B7-H3 expression (negative or positive) in strata of pathological stage. (**C**) Pathological stage I−II. (**D**) Pathological stage III−IV.

## Abbreviations

CI	confidence interval
DFS	disease-free survival
HR	hazard ratio
JFCR	Japanese Foundation for Cancer Research
NSCLC	non-small-cell lung cancer
p-stage	pathological stage
TMA	tissue microarray

## References

[1] Siegel R.L., Miller K.D., Jemal A. (2017). Cancer Statistics, 2017. CA Cancer J. Clin..

[2] Ryan D.P., Hong T.S., Bardeesy N. (2014). Pancreatic adenocarcinoma. N. Engl. J. Med..

[3] Maitra A., Hruban R.H. (2008). Pancreatic cancer. Annu. Rev. Pathol..

[4] Vonderheide R.H., Bayne L.J. (2013). Inflammatory networks and immune surveillance of pancreatic carcinoma. Curr. Opin. Immunol..

[5] Orozco C.A., Martinez-Bosch N., Guerrero P.E., Vinaixa J., Dalotto-Moreno T., Iglesias M., Moreno M., Djurec M., Poirier F., Gabius H.J. (2018). Targeting galectin-1 inhibits pancreatic cancer progression by modulating tumor-stroma crosstalk. Proc. Natl. Acad. Sci. USA.

[6] Xu J.W., Wang L., Cheng Y.G., Zhang G.Y., Hu S.Y., Zhou B., Zhan H.X. (2018). Immunotherapy for pancreatic cancer: A long and hopeful journey. Cancer Lett..

[7] Wartenberg M., Cibin S., Zlobec I., Vassella E., Eppenberger-Castori S.M.M., Terracciano L., Eichmann M., Worni M., Gloor B., Perren A. (2018). Integrated genomic and immunophenotypic classification of pancreatic cancer reveals three distinct subtypes with prognostic/predictive significance. Clin. Cancer Res..

[8] Bishehsari F., Zhang L., Barlass U., Preite N., Turturro S., Najor M.S., Shetuni B.B., Zayas J.P., Mahdavinia M., Abukhdeir A.M. (2018). KRAS Mutation and Epithelial-Macrophage Interplay in Pancreatic Neoplastic Transformation. Int. J. Cancer.

[9] Veenstra V.L., Garcia-Garijo A., van Laarhoven H.W., Bijlsma M.F. (2018). Extracellular Influences: Molecular Subclasses and the Microenvironment in Pancreatic Cancer. Cancers (Basel).

[10] Mei L., Du W., Ma W.W. (2016). Targeting stromal microenvironment in pancreatic ductal adenocarcinoma: controversies and promises. J. Gastrointest. Oncol..

[11] Forde P.M., Chaft J.E., Smith K.N., Anagnostou V., Cottrell T.R., Hellmann M.D., Zahurak M., Yang S.C., Jones D.R., Broderick S. (2018). Neoadjuvant PD-1 Blockade in Resectable Lung Cancer. N. Engl. J. Med..

[12] Tie Y., Ma X., Zhu C., Mao Y., Shen K., Wei X., Chen Y., Zheng H. (2017). Safety and efficacy of nivolumab in the treatment of cancers: A meta-analysis of 27 prospective clinical trials. Int. J. Cancer.

[13] Martinez-Bosch N., Vinaixa J., Navarro P. (2018). Immune Evasion in Pancreatic Cancer: From Mechanisms to Therapy. Cancers (Basel).

[14] Zhang J., Wolfgang C.L., Zheng L. (2018). Precision Immuno-Oncology: Prospects of Individualized Immunotherapy for Pancreatic Cancer. Cancers (Basel).

[15] Foley K., Kim V., Jaffee E., Zheng L. (2016). Current progress in immunotherapy for pancreatic cancer. Cancer Lett..

[16] Brahmer J.R., Tykodi S.S., Chow L.Q., Hwu W.J., Topalian S.L., Hwu P., Drake C.G., Camacho L.H., Kauh J., Odunsi K. (2012). Safety and activity of anti-PD-L1 antibody in patients with advanced cancer. N. Engl. J. Med..

[17] Royal R.E., Levy C., Turner K., Mathur A., Hughes M., Kammula U.S., Sherry R.M., Topalian S.L., Yang J.C., Lowy I. (2010). Phase 2 trial of single agent Ipilimumab (anti-CTLA-4) for locally advanced or metastatic pancreatic adenocarcinoma. J. Immunother..

[18] Balachandran V.P., Luksza M., Zhao J.N., Makarov V., Moral J.A., Remark R., Herbst B., Askan G., Bhanot U., Senbabaoglu Y. (2017). Identification of unique neoantigen qualities in long-term survivors of pancreatic cancer. Nature.

[19] Wang L., Kang F.B., Shan B.E. (2014). B7-H3-mediated tumor immunology: Friend or foe?. Int. J. Cancer.

[20] Chapoval A.I., Ni J., Lau J.S., Wilcox R.A., Flies D.B., Liu D., Dong H., Sica G.L., Zhu G., Tamada K. (2001). B7-H3: a costimulatory molecule for T cell activation and IFN-gamma production. Nat. Immunol..

[21] Sun M., Richards S., Prasad D.V., Mai X.M., Rudensky A., Dong C. (2002). Characterization of mouse and human B7-H3 genes. J. Immunol..

[22] Yonesaka K., Haratani K., Takamura S., Sakai H., Kato R., Takegawa N., Takahama T., Tanaka K., Hayashi H., Takeda M. (2018). B7-H3 Negatively Modulates CTL-Mediated Cancer Immunity. Clin. Cancer Res..

[23] Marmarelis M.E., Aggarwal C. (2018). Combination Immunotherapy in Non-small Cell Lung Cancer. Curr. Oncol. Rep..

[24] Marin-Acevedo J.A., Dholaria B., Soyano A.E., Knutson K.L., Chumsri S., Lou Y. (2018). Next generation of immune checkpoint therapy in cancer: new developments and challenges. J. Hematol. Oncol..

[25] Ignatiadis M., Van den Eynden G., Roberto S., Fornili M., Bareche Y., Desmedt C., Rothe F., Maetens M., Venet D., Holgado E. (2018). Tumor-Infiltrating Lymphocytes in Patients Receiving Trastuzumab/Pertuzumab-Based Chemotherapy: A TRYPHAENA Substudy. Natl. Cancer Inst..

[26] Seaman S., Zhu Z., Saha S., Zhang X.M., Yang M.Y., Hilton M.B., Morris K., Szot C., Morris H., Swing D.A. (2017). Eradication of Tumors through Simultaneous Ablation of CD276/B7-H3-Positive Tumor Cells and Tumor Vasculature. Cancer Cell.

[27] Burugu S., Dancsok A.R., Nielsen T.O. (2017). Emerging targets in cancer immunotherapy. Semin. Cancer Biol..

[28] Picarda E., Ohaegbulam K.C., Zang X. (2016). Molecular Pathways: Targeting B7-H3 (CD276) for Human Cancer Immunotherapy. Clin. Cancer Res..

[29] Yamato I., Sho M., Nomi T., Akahori T., Shimada K., Hotta K., Kanehiro H., Konishi N., Yagita H., Nakajima Y. (2009). Clinical importance of B7-H3 expression in human pancreatic cancer. Br. J. Cancer.

[30] Loos M., Hedderich D.M., Ottenhausen M., Giese N.A., Laschinger M., Esposito I., Kleeff J., Friess H. (2009). Expression of the costimulatory molecule B7-H3 is associated with prolonged survival in human pancreatic cancer. BMC Cancer.

[31] Li D., Wang J., Zhou J., Zhan S., Huang Y., Wang F., Zhang Z., Zhu D., Zhao H., Li D. (2017). B7-H3 combats apoptosis induced by chemotherapy by delivering signals to pancreatic cancer cells. Oncotarget.

[32] Xu H., Chen X., Tao M., Chen K., Chen C., Xu G., Li W., Yuan S., Mao Y. (2016). B7-H3 and B7-H4 are independent predictors of a poor prognosis in patients with pancreatic cancer. Oncol. Lett..

[33] Chen Y., Sun J., Zhao H., Zhu D., Zhi Q., Song S., Zhang L., He S., Kuang Y., Zhang Z. (2014). The coexpression and clinical significance of costimulatory molecules B7-H1, B7-H3, and B7-H4 in human pancreatic cancer. OncoTargets Ther..

[34] Xu L., Ding X., Tan H., Qian J. (2013). Correlation between B7-H3 expression and matrix metalloproteinases 2 expression in pancreatic cancer. Cancer Cell Int..

[35] Zhang X., Fang C., Zhang G., Jiang F., Wang L., Hou J. (2017). Prognostic value of B7-H3 expression in patients with solid tumors: a meta-analysis. Oncotarget.

[36] Amin M.B., Edge S.B., Greene F.L., Byrd D.R., Brookland R.K., Washington M.K., Gershenwald J.E., Compton C.C., Hess K.R., Sullivan D.C. (2017). AJCC Cancer Staging Manual.

[37] Hiramatsu M., Ninomiya H., Inamura K., Nomura K., Takeuchi K., Satoh Y., Okumura S., Nakagawa K., Yamori T., Matsuura M. (2010). Activation status of receptor tyrosine kinase downstream pathways in primary lung adenocarcinoma with reference of KRAS and EGFR mutations. Lung Cancer.

[38] Inamura K., Yokouchi Y., Kobayashi M., Sakakibara R., Ninomiya H., Subat S., Nagano H., Nomura K., Okumura S., Shibutani T. (2017). Tumor B7-H3 (CD276) expression and smoking history in relation to lung adenocarcinoma prognosis. Lung Cancer.

[39] Inamura K., Yokouchi Y., Sakakibara R., Kobayashi M., Subat S., Ninomiya H., Nagano H., Nomura K., Okumura S., Ishikawa Y. (2016). Relationship of tumor PD-L1 expression with EGFR wild-type status and poor prognosis in lung adenocarcinoma. Jpn. J. Clin. Oncol..

[40] Pylayeva-Gupta Y., Lee K.E., Hajdu C.H., Miller G., Bar-Sagi D. (2012). Oncogenic Kras-induced GM-CSF production promotes the development of pancreatic neoplasia. Cancer Cell.

[41] Vigdorovich V., Ramagopal U.A., Lazar-Molnar E., Sylvestre E., Lee J.S., Hofmeyer K.A., Zang X., Nathenson S.G., Almo S.C. (2013). Structure and T cell inhibition properties of B7 family member, B7-H3. Structure.

[42] Zhao X., Li D.C., Zhu X.G., Gan W.J., Li Z., Xiong F., Zhang Z.X., Zhang G.B., Zhang X.G., Zhao H. (2013). B7-H3 overexpression in pancreatic cancer promotes tumor progression. Int. J. Mol. Med..

[43] Ingebrigtsen V.A., Boye K., Nesland J.M., Nesbakken A., Flatmark K., Fodstad O. (2014). B7-H3 expression in colorectal cancer: associations with clinicopathological parameters and patient outcome. BMC Cancer.

[44] Kraan J., Van den Broek P., Verhoef C., Grunhagen D.J., Taal W., Gratama J.W., Sleijfer S. (2014). Endothelial CD276 (B7-H3) expression is increased in human malignancies and distinguishes between normal and tumour-derived circulating endothelial cells. Br. J. Cancer.

