# Supplementary material for: Tumor B7-H3 (CD276) Expression and Survival in Pancreatic Cancer
Source: J Clin Med. 2018 Jul 10;7(7):172. doi: 10.3390/jcm7070172 (PMC6069252; doi:10.3390/jcm7070172)
Supplement: Supplementary file 1 [file jcm-07-00172-s001.pdf]

Supplementary Materials: Tumor B7-H3 (CD276) Expression and Survival in Pancreatic Cancer

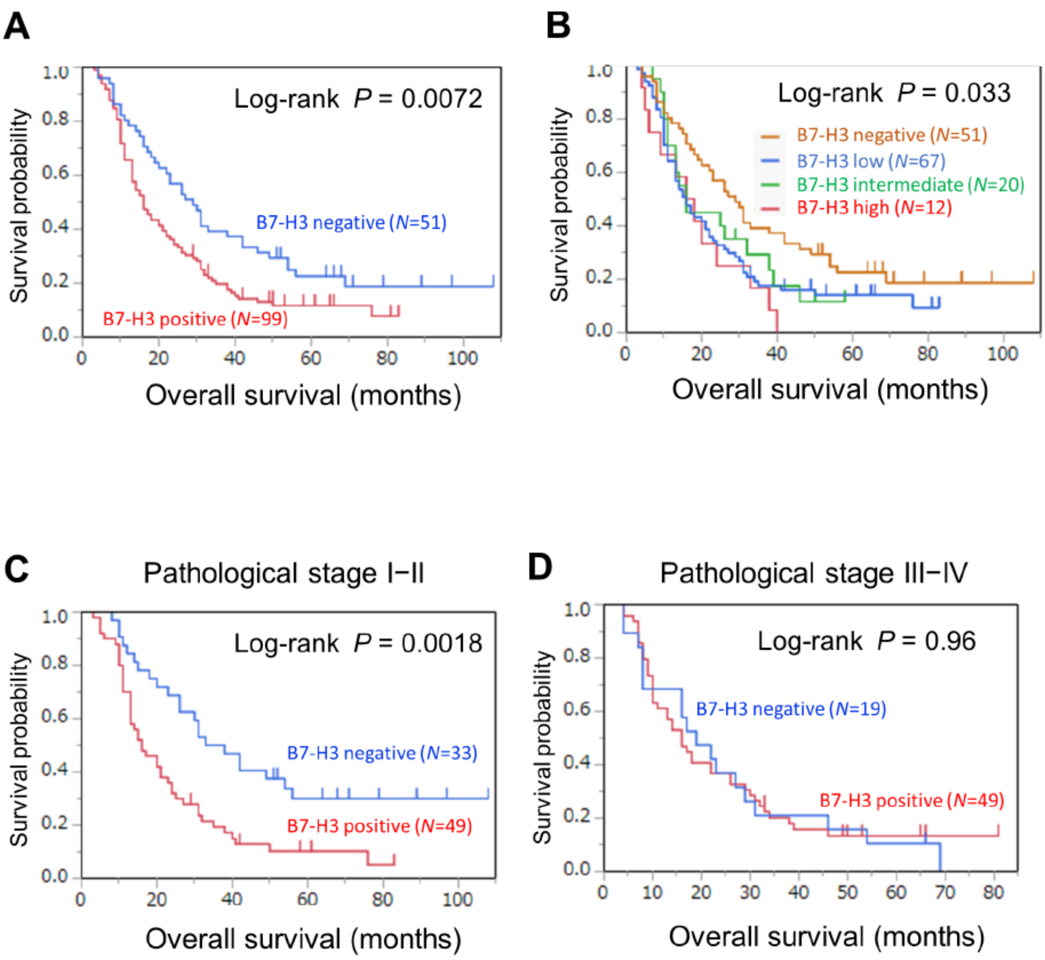

Figure S1. The following is available online at www.mdpi.com/link, Figure S1: Kaplan-Meier curves for overall survival in patients with pancreatic cancer (**A**) according to tumor B7-H3 expression (negative or positive) or (**B**) according to tumor B7-H3 expression (negative, low, intermediate, or high). Kaplan-Meier curves for overall survival in patients with pancreatic cancer according to tumor B7-H3 expression (negative or positive) in strata of pathological stage. (**C**) Pathological stage Ⅰ−Ⅱ. (**D**) Pathological stage Ⅲ−Ⅳ.
